# Improving Consumer Satisfaction with Addiction Treatment: An Analysis of Alumni Preferences

**DOI:** 10.1155/2015/509864

**Published:** 2015-09-21

**Authors:** Ruchi M. Sanghani, Alexander K. Moler

**Affiliations:** Recovery Brands, New York, NY 10003, USA

## Abstract

*Objective*. The primary objective of this investigation is to determine which individual and aggregate factors of residential addiction treatment centers are most significant influencers of alumni satisfaction. *Design*. Survey targeted alumni of residential addiction treatment facilities. Alumni were queried through a survey, which utilized Likert-scale matrices and binary response options: 379 respondents met the completion threshold. Alumni rated amenities and individual and group counseling factors; additionally, respondents provided feedback on two satisfaction proxies: cost worthiness and future recommendations. Descriptive and relational analyses were conducted, with the latter utilizing logistic regression models. *Results*. Individual factors' scores of group counseling, and overall aggregate group counseling score, are most enthusiastically positive. Group counseling is also the most significant influencer of satisfaction. Other significant influencers of satisfaction are met expectations for individual counseling and psychiatric care offerings. *Conclusions*. While individual counseling and facility amenities should not be ignored, group counseling may be the most significant influencer of alumni satisfaction. Long-term outcomes are not single-faceted; however, treatment providers should be encouraged to invest in high-quality group counseling offerings in order to best satisfy, and thereby empower, clients.

## 1. Introduction

Consumer satisfaction is essential for any business [1]. Businesses offering goods and services to consumers often request feedback from former customers to assess strengths and weaknesses and to leverage positive feedback and testimonials as marketing materials. As these testimonials become more digitized and more public on sites like Yelp, Facebook, and Google+, businesses have a high stake in ensuring that their consumers are satisfied with the quality of service or goods they provide [2, 3].

In the healthcare space, patient satisfaction has been formally assessed for several years. The Hospital Consumer Assessment of Healthcare Providers and Systems (HCAHPS) initiative provides a survey tool to measure patients' perspectives and satisfaction within hospital systems [4]. The HCAHPS survey aims to produce standardized data on patients' perspectives on hospital care to allow for meaningful comparisons between hospitals based on consumer importance and publicly represent these findings to encourage transparency and incentivize hospital systems to improve their quality of care [4].

The HCAHPS initiative has identified 21 rating items that have been determined to affect patient perspectives on hospital care and has generated a 32-question survey that assesses quality of care from nurses and doctors, hospital environment, and experiences in the hospital. While this survey instrument is widely used in the hospital setting, an equivalent tool does not exist to evaluate satisfaction and perspectives in other areas of medicine and health, including addiction rehabilitation programs. Unique in its function, residential addiction treatment centers simultaneously operate as healthcare providers and hospitality managers, delivering medical and psychological support while offering accommodations, meals, and amenities in lieu of a clients' typical home environment.

In this exploratory study, we aim to determine which criteria are most significant to influencing overall alumni satisfaction with an inpatient drug or alcohol rehabilitation facility. Because the residential and intensive outpatient addiction rehabilitation industry functions at the crux of social science, medicine, and hospitality services, we hypothesize that alumni perceptions of facility amenities and perceived quality of individual therapies and group counseling will have significant influence on alumni satisfaction. We also hypothesize that the presence of subsequent relapse after discharge from a facility as a proxy measurement for success will negatively influence alumni satisfaction.

## 2. Methods

### 2.1. Survey Design

A survey was created using SurveyMonkey's online survey design platform to assess alumni feedback and satisfaction of a recent stay in an inpatient drug or alcohol rehabilitation facility [5]. The survey contains 45 questions with an equal mix of single choice and open-ended questions. The survey also includes three matrix questions for evaluation of facility features, individual therapy, and group counseling. This survey is hosted on Rehabs.com's website [6].

Respondents were asked to evaluate their facilities by the following criteria: the facility's amenities and individual and group counseling services. They were also asked to respond to demographic and personal questions regarding age of entry into treatment, method of payment, and motivation to enter treatment. Responses to the facility services questions were collected on a 5-Point Likert Scale. The evaluated aspects are as follows:

- *amenities*—accommodations, meals and nutrition, exercise options, leisure/extracurricular, holistic offerings (e.g., yoga and meditation), staff support, connectivity (Internet/phone use), visitor policy, and cleanliness;
- *individual counseling*—counselor availability, counselor training/experience, counselor respect for patient's treatment preferences, flexibility to switch counselors, inclusion of holistic approaches, and inclusion of alternative/creative treatment approaches;
- *group counseling*—quality of lead counselor, frequency of meetings, consistency of meetings, member empowerment/safety, and conflict resolution.

### 2.2. Sample Selection

The survey targets alumni of inpatient drug or alcohol rehabilitation facilities whose most recent enrollment occurred within the past ten years. Respondents were targeted through SurveyMonkey Audience, which is a service that provides survey takers with targeted, representative sample populations [5]. Additionally, respondents were asked a qualifying question, to ensure they met targeting requirements. Inclusion criteria for analyses include a minimum survey completion of 75% over the variables forming the aggregate scores (i.e., category components) and 100% over the remaining relevant variables. Survey data collected from August 25, 2014, to February 22, 2015, were included in these analyses.

### 2.3. Analytical Approach

Survey data were cleaned, recoded, and analyzed using Stata/IC 13.1 [7]. Of the 864 survey responses received, 485 responses did not fit the sample parameters for completeness and were removed from the database, resulting in a sample of 379 surveys. Recoding was required to convert the survey platform's default coding into a consistent format compatible with Stata's requirements. Full details regarding cleaning and coding (.log) are available at the reader's request.

Data were analyzed using a variety of logistic regression models, due to the outcome variables being dichotomous. For the purpose of these analyses, the dataset was declared as survey data, using Stata's svyset command. Each respondent served as a sampling unit. Linearized variance estimation was used. All of the following analyses utilized the appropriate svy command prefix, unless otherwise noted.

The likelihood of a respondent responding positively (i.e., answering “yes”) to the binary questions “was treatment worth the cost?” and “would you recommend the facility to a friend or family member?” was assessed by examining the impact of the respondent's perception of the facility's offerings. To better evaluate this potential impact, the individual offerings in each category were condensed into three aggregate variables. Each aggregate variable was formed by finding the mean of the respective category component scores for each respondent.

All data were deidentified, and respondents provided consent for use of survey data in public reporting and research directly on the survey.

## 3. Results

Results from 379 surveys met inclusion criteria and were used for analyses. Two metrics were considered indicators of satisfaction with the facility attended: if treatment was worth the cost, and if the respondent would recommend this facility to a friend or family member who is seeking addiction treatment. These two metrics were considered indicators of satisfaction as recommendations and personal cost-benefit ratios are standard proxies for satisfaction in marketing practices, as well as in health service assessments [4, 8–14].

### 3.1. Survey Population Profile and Facility Satisfaction

#### 3.1.1. Population Profile

Detailed information regarding demographic information can be found in Table 1. 66.05% (*n* = 249) of respondents entered inpatient addiction treatment between 40 and 59 years of age. Treatment was sought by individuals most frequently at their own behest (62.33%  *n* = 235) and was most commonly paid for through private (33.86%  *n* = 128) or government-provided (18.25%  *n* = 69) insurance.

Cross tabulations were also conducted to view the intersection of these entrance factors. The most frequently occurring profile is an individual that entered treatment as a personal choice between the ages of 40 and 59 years. This profile fits 42.13% (*n* = 158) of respondents. The second most frequently occurring profile is an individual that entered treatment as a personal choice, between the ages of 26 and 39 years; this profile fits 13.87% (*n* = 52) of respondents.

#### 3.1.2. Perception of Facilities

Likert-scale scores were calculated for each individual metric regarding amenities, individual counseling, and group counseling (Table 2). Amenities received an overall composite Likert-scale score of 3.99 (sd = 0.97). Respondents scored four out of nine amenities factors highly (Likert-scale score > 4.00). With regard to individual counseling offerings, which received an overall score of 3.84 (sd = 1.14), respondents highly rated counselor training and experience (4.36 sd = 1.10), as well as their respect for the individual's treatment preferences (4.25 sd = 1.18). Respondents were less pleased with counselors' willingness to incorporate holistic approaches (3.18 sd = 1.62). Group counseling offerings were given an overall score of 4.37 (sd = 1.00). Each of the five factors of group counseling was given a score greater than 4.00, with the frequency of group meetings receiving the highest Likert-scale score.

#### 3.1.3. Was Treatment Worth the Cost?

Four models were constructed using logistic regression to evaluate factors impacting an individual's notion of treatment being worth the cost. The results from these models are found in Table 3. The first model regresses the three summed aggregate offering scores regarding amenities (variable name = afacilityscore), individual counseling (acounsscore), and group counseling (agrpcounsscore) on the variable for “was treatment worth the cost?” (worcost). The second model regresses three “expectation” variables on worcost. These variables are “did the facility deliver the promised amount of counseling?” (promcouns); “did psychiatric care meet expectations?” (psyexpect); and “did marketing materials accurately portray the facility's offerings?” (market). The third model regresses the preceding six models on worcost. The fourth model adjusts model 1c, taking into account whether or not the respondent had relapsed. The results of each model are as follows:

(i) *Model 1a*—the aggregate mean group score is statistically significant, with a *p* value of 0.022. The group counseling score produced the highest degree of substantive significance; each point increase of the group counseling score corresponds with an increase in the odds of the respondent regarding their treatment as worth the cost by 126%.
(ii) *Model 1b*—all three variables are statistically significant. Whether or not the facility provided the promised amount of counseling has the highest degree of substantive significance. Moving from “no” to “yes” on this variable corresponds to approximately a 37-fold increase in the odds of the respondent regarding his treatment as worth the cost.
(iii) *Model 1c*—combining the previous two models yields interesting results. Notably, when adjusting for offerings scores, the marketing materials accuracy becomes statistically insignificant. Additionally, “psychiatric care” is no longer significant to the 0.001 level; it also loses degrees of substantive significance. When taken together, this suggests that these variables are not the strongest predictive components. Promised amount of counseling and aggregate mean group counseling score remain statistically significant and increase in substantive significance.
(iv) *Model 1d*—model suggests that “relapse” is not a predictive component of whether or not the respondent considers their treatment as worth the cost, as “relapse” is not statistically significant. Furthermore, adjusting for relapse increases the substantive significance of aggregate mean group counseling score and “psychiatric expectations” score. Reproducing this model without the svy-estimation and conducting a Likelihood ratio test with 1c yield a statistically insignificant result, suggesting that the model is not an improvement over 1c.

#### 3.1.4. Would You Recommend the Facility?

Four models were constructed using logistic regression to determine potentially influential factors on alumni's willingness to recommend the facility where they received treatment, represented by the variable recff. The results from these models are found in Table 4. The first model regresses the aforementioned three aggregate mean offering scores (afacilityscore, acounsscore, and agrpcounsscore) on recff. The second model regresses the three aforementioned “expectation” variables (promcouns, psyexpect, and market) on recff. The third model regresses the preceding six variables on recff. The results of each model are as follows:

- *Model 2a*—each point increase of the group counseling score corresponds with an increase in the odds of the respondent recommending the facility by 256%. The other two variables are not statistically significant.
- *Model 2b*—all three included variables are significant to, at least, the 0.001 level. Of these variables, the most influential is the variable representing the promised amount of counseling; a respondent answering “yes” to this question corresponds to an increase in the odds that they would recommend the facility approximately 661%.
- *Model 2c*—by increasing model specification, a clearer picture of recff's predictors becomes apparent. For the group offering score and promised amount of counseling, the relative consistency in odds ratios and statistical significance across the two models suggests that these variables are influential factors in a facility receiving a positive alumni recommendation. This model also suggests that the respondent's perceptions of the facility's amenity offerings and individual counseling are not predictor variables, as these variables produced a statistically insignificant output over both models. Finally, when controlling for the offerings scores, the psychiatric expectation and accuracy of marketing materials variables are not statistically significant, suggesting that they may not be strong predictor variables, despite being statistically significant predictors in model 2b.
- *Model 2d*—“relapse” is not a significant predictor. Adjusting for relapse has minimal substantive effects on the significant predictor variables. As with 1d, reproducing this model without the svy-estimation and conducting a Likelihood ratio test with 2c yield a statistically insignificant result, suggesting the model is not an improvement over 2c.

## 4. Discussion

The respondents' evaluations of their respective facility's offerings shed insight into the population's perception of inpatient treatment. The study results indicate trends in facility offerings and respondents' perceptions. In the aggregate, facilities are generally regarded as providing satisfactory to highly satisfactory offerings. This finding is consistent with reviews across other industries, where the literature has shown that the general reviews tend to have a negative skew distribution [15]. Alumni most highly rated group counseling. Alumni reception towards facilities' amenities and individual counseling is generally positive, albeit to a less enthusiastic degree compared to group counseling. Facilities' individual counseling offerings were the least enthusiastically received, particularly in regard to the facilities' inclusion of holistic, alternative, and creative approaches. This dissatisfaction with holistic offerings is also present in the amenities category, indicating that such an attitude is pervasive and present in the respondents' overall perception of the facility.

These analyses demonstrate important factors that may influence alumni satisfaction. Models 1a–d aim to determine the most substantial predictors of satisfaction with the financial value provided by treatment. These models suggest that high-quality group counseling, as well as meeting or exceeding expectations for the amount of counseling received, is the most substantial and substantively significant predictors of satisfaction relating to cost worthiness. Additional statistically significant predictors of satisfaction for this metric are psychiatric care meeting expectations and, to a lesser extent, individual counseling offerings. Models 2a–d suggest key factors in alumni's willingness to recommend a facility to their friends or family members. Specifically, the results suggest that the respondent's perceptions of their group counseling experiences, whether or not the facility delivered its promised amount of counseling and, to a slightly lesser extent, whether psychiatric care meets expectations, are instrumental in alumni's decision to recommend their facility.

The selected outcome variables and their responses are proxy measures of consumer satisfaction with treatment facilities' services. Evaluating the models related to these two variables simultaneously highlights strong and consistent potential predictors of consumer satisfaction with treatment facilities' services. For both outcomes, and across each relevant model, the aggregate group counseling score and whether the facility kept its promises regarding individual counseling are statistically significant predictors of a respondent's (i.e., consumer's) satisfaction with his or her treatment. This consistency across outcomes and models suggests the presence of predictive power for these two aspects of treatment services. To a lesser extent, this consistency is also observed for variables representing individual counseling and whether the facility delivered the expected psychiatric care.

These results are strengthened by the failure to confirm the second hypothesis; adjusted for the other selected variables, whether the respondent had relapsed or not was not a statistically significant predictor of alumni satisfaction. Furthermore, its inclusion with these other potential predictor variables may not serve as necessary or beneficial specification. Facilities should not approach their clients' satisfaction with fatalistic uncertainty that the client's postdischarge actions will solely create a negative retrospective perception of treatment; rather, facilities should recognize that their actions can directly and primarily impact satisfaction, regardless of long-term client outcomes.

Improving offerings and, in turn, improving satisfaction can aid recovery. It is well documented in the literature that higher patient satisfaction scores correlate well with medical treatment adherence [16, 17]. For those struggling with addiction, adherence to a treatment plan is vital to recovery. Facilities should aim to provide the most satisfactory service to promote treatment adherence. While individual counseling and amenity offerings certainly should not be ignored, high-quality group counseling should be supported and promoted to enhance clients' treatment experience. Improving satisfaction with group counseling offerings has the highest likelihood of improving overall consumer satisfaction, which in turn is likely to support greater adherence to a recovery plan—a goal that should guide facilities' operations. Through incorporating empirical research into operational policy, facilities can ensure that changes they make have a demonstrable impact.

The importance placed on group counseling offerings should not solely be viewed through the lens of predicting consumer satisfaction, as broader implications for the treatment space exist. In addition to the aggregate group counseling score being the most consistent substantively and statistically significant predictor, each of its components, collectively, had the highest mean perception scores of the three aggregate categories. Viewed together, these findings suggest that consumers are most enthusiastic and receptive to group counseling offerings.

Investigations into the role of social support on treatment adherence within the addiction space, and across several other health and wellness industries, have demonstrated moderate to significant improvements in adherence [18–21]. Just as satisfaction can improve adherence, adherence—and subsequently resulting treatment “success”—can positively influence retrospective perceived satisfaction, thus beginning a cyclical pattern for satisfaction and treatment adherence [22, 23]. For treatment providers, the construction of social support during treatment may build the foundation for successful and satisfactory long-term recovery. Future research should aim to determine how group therapies improve satisfaction scores and treatment adherence to encourage clients' recovery.

This study includes the limitation that only two metrics were chosen as proxies for satisfaction. While cost worthiness and recommendations are significant proxies for satisfaction, other proxies may also exist. Subsequent investigations may include the use of other metrics indicating satisfaction to test our findings. Respondent recruitment utilized a self-selection method, which could contribute to selection bias within the study's sample population. Specifically, as the survey was hosted online, respondent demographics will reflect general Internet user demographics, which may not be reflective of the general population. Precise demographics for both the general population of rehabilitation alumni are unavailable, limiting the ability to determine sample representativeness. Additionally, as reviews tend to skew positive, the potential predictor factors discussed below may not be generalizable for unenthusiastic alumni. Selection bias will be further addressed as future studies move away from exploratory efforts.

## 5. Conclusions

Through producing multiple models in regard to two outcome variables, our results highlight potentially key predictors of alumni's satisfaction with their treatment experience. Across both outcome variables, alumni's perceptions of the facility's group offerings and the facility keeping its promises regarding individual counseling are statistically and substantively significant. The alumni's perceptions of the facility's marketing materials' accuracy and whether the facility delivered the expected psychiatric care are also statistically and substantively significant, but inconsistently so. Satisfaction with treatment may be correlated with increased treatment adherence and subsequent long-term success in recovery. The addiction treatment space would benefit greatly from further research into the impact of these predictors, through refining of the aggregate component measurements, as well as evaluating the impact of components themselves, to gain a more clear understanding of predictors of consumer satisfaction and thus a better understanding of the treatment space.

## Figures and Tables

**Table 1:** Population profile of 379 survey respondents.

Demographic category	Sample size	Frequency	Percent distribution
Age of entering treatment	377		
Under 18		1	0.27
18–25		20	5.31
26–39		86	22.81
40–59		249	66.05
60+		21	5.57
Reason for entering treatment	377		
Family intervention		53	14.06
Court mandated		23	6.10
Doctor recommended		36	9.55
Personal choice		235	62.33
Other		30	7.96
Referral to facility	379		
Medical referral		87	22.96
Friend or family referral		118	31.13
Legal referral		16	4.22
Personal search		101	26.65
Other		57	15.04
Primary payment method	378		
Self-pay		60	15.87
Family or friend support		23	6.08
External financing (e.g., loan)		2	0.53
State or federal insurance		69	18.25
Private insurance		128	33.86
Combination of the above		49	12.96
Other		47	12.43

**Table 2:** Mean values for respondent's ratings of facility offerings.

Facility offering	Sample size	Mean	Standard deviation
Amenity category			
Accommodations	378	4.23	1.03
Meals and nutrition	368	4.39	1.01
Exercise options	366	3.81	1.27
Leisure and extracurricular offerings	259	3.80	1.27
Holistic offerings	319	3.19	1.60
Staff support	367	4.38	1.12
Connectivity	338	3.48	1.49
Visitor policy	355	3.98	1.31
Cleanliness	366	4.46	1.01
Individual counseling category			
Counselor availability	364	3.95	1.34
Counselor training and experience	375	4.36	1.10
Respect for patient's treatment preferences	370	4.25	1.18
Flexibility to switch counselors	299	3.58	1.54
Inclusion of holistic approaches	308	3.18	1.62
Inclusion of alternative approaches	324	3.34	1.58
Group counseling category			
Quality of lead counselor	378	4.38	1.08
Frequency of meetings	378	4.48	0.99
Consistency of meetings	378	4.42	1.03
Member empowerment and safety	369	4.32	1.15
Conflict resolution	371	4.23	1.19
Composite scores			
Aggregate mean amenity score	379	3.99	0.97
Aggregate mean individual counseling score	379	3.84	1.14
Aggregate mean group counseling score	379	4.37	1.00

**Table 3:** Regression outcomes (worcost).

Model	Dependent variables	Sample size	Odds ratio	*T*-statistic	*p* value	95% confidence interval
1a	Aggregate mean amenity score	379	1.73	1.15	0.252	0.68–4.43
	Aggregate mean individual counseling score		1.55	1.35	0.177	0.82–2.94
	Aggregate mean group counseling score		2.26	2.30	0.022	1.13–4.55

1b	Promised amount of counseling	379	37.40	7.31	<0.001	14.13–99.01
	Psychiatric care meeting expectations		6.62	3.94	<0.001	2.58–17.02
	Marketing materials accuracy		3.13	2.28	0.023	1.17–8.41

1c	Aggregate mean amenity score	379	1.92	1.77	0.078	0.93–3.96
	Aggregate mean individual counseling score		0.58	−1.24	0.217	0.25–1.38
	Aggregate mean group counseling score		2.65	2.29	0.023	1.15–6.13
	Promised amount of counseling		45.01	5.61	<0.001	11.86–170.78
	Psychiatric care meeting expectations		3.41	2.32	0.021	1.20–9.64
	Marketing materials accuracy		0.92	−0.015	0.883	0.30–2.85

1d^*∗*^	Aggregate mean amenity score	379	1.84	1.48	0.141	0.82–4.12
	Aggregate mean individual counseling score		0.60	−1.23	0.218	0.26–1.36
	Aggregate mean group counseling score		2.69	2.22	0.027	1.12–6.47
	Promised amount of counseling		44.88	5.59	<0.001	11.77–171.08
	Psychiatric care meeting expectations		3.51	2.23	0.026	1.16–10.65
	Marketing materials accuracy		0.93	−0.13	0.899	0.30–2.90
	Respondent relapsed		1.20	0.32	0.749	0.392–3.68

^*∗*^Likelihood ratio test 1d/1c not significant on non-svy (*p* = 0.760).

**Table 4:** Regression outcomes (recff).

Model	Dependent variables	Sample size	Odds ratio	*T*-statistic	*p* value	95% confidence interval
2a	Aggregate mean amenity score	379	1.55	0.87	0.387	0.57–4.16
	Aggregate mean individual counseling score		1.48	1.12	0.262	0.75–2.92
	Aggregate mean group counseling score		2.56	2.79	0.006	1.32–4.97

2b	Promised amount of counseling	379	7.61	3.80	<0.001	2.67–21.76
	Psychiatric care meeting expectations		4.89	3.50	0.001	2.00–11.91
	Marketing materials accuracy		6.43	3.22	0.001	2.06–20.04

2c	Aggregate mean amenity score	379	1.20	0.43	0.669	0.52–2.80
	Aggregate mean individual counseling score		1.05	0.11	0.909	0.48–2.30
	Aggregate mean group counseling score		2.40	3.19	0.002	1.40–4.12
	Promised amount of counseling		4.65	2.51	0.012	1.40–15.45
	Psychiatric care meeting expectations		2.47	1.76	0.079	0.90–6.75
	Marketing materials accuracy		2.52	1.52	0.130	0.76–8.38

2d^*∗*^	Aggregate mean amenity score	379	1.36	0.68	0.495	0.56–3.32
	Aggregate mean individual counseling score		0.94	−0.16	0.876	0.40–2.17
	Aggregate mean group counseling score		2.37	3.08	0.002	1.37–4.11
	Promised amount of counseling		4.95	2.61	0.009	1.49–16.51
	Psychiatric care meeting expectations		2.31	1.59	0.112	0.82–6.55
	Marketing materials accuracy		2.48	1.48	0.141	0.74–8.30
	Respondent relapsed		0.56	−1.11	0.268	0.20–1.57

^*∗*^Likelihood ratio test 1d/1c not significant on non-svy (*p* = 0.235).
